# Detection of Upscale-Crop and Partial Manipulation in Surveillance Video Based on Sensor Pattern Noise

**DOI:** 10.3390/s130912605

**Published:** 2013-09-18

**Authors:** Dai-Kyung Hyun, Seung-Jin Ryu, Hae-Yeoun Lee, Heung-Kyu Lee

**Affiliations:** 1 Department of Computer Science, Korea Advanced Institute of Science and Technology, 291 Daehak-Ro, Yuseong-Gu, Daejon, Korea; E-Mails: dkhyun@mmc.kaist.ac.kr (D.-K.H.); ryu.seungjin@gmail.com (S.-J.R.); 2 Department of Computer and Software Engineering, Kumoh National Institute of Technology, Yangho-dong, Gumi, Gyeongbuk, Korea; E-Mail: haeyeoun.lee@kumoh.ac.kr

**Keywords:** digital image forensic, sensor pattern noise, forgery detection, surveillance video forgery, MACE-MRH correlation filter

## Abstract

In many court cases, surveillance videos are used as significant court evidence. As these surveillance videos can easily be forged, it may cause serious social issues, such as convicting an innocent person. Nevertheless, there is little research being done on forgery of surveillance videos. This paper proposes a forensic technique to detect forgeries of surveillance video based on sensor pattern noise (SPN). We exploit the scaling invariance of the minimum average correlation energy Mellin radial harmonic (MACE-MRH) correlation filter to reliably unveil traces of upscaling in videos. By excluding the high-frequency components of the investigated video and adaptively choosing the size of the local search window, the proposed method effectively localizes partially manipulated regions. Empirical evidence from a large database of test videos, including RGB (Red, Green, Blue)/infrared video, dynamic-/static-scene video and compressed video, indicates the superior performance of the proposed method.

## 1. Introduction

With the development of highly sophisticated imaging devices, digital images are commonly used in our ordinary lives. The benefits of digital images, such as facility in editing, sharing and storing, motivate people to use digital images instead of analog images. At the same time, the very nature of digital data, which can be easily manipulated, encourages abuse of the digital images; hence, the authentication of images has become one of the biggest issues regarding digital images.

Digital image forensics, which detects modification of digital images, has been actively researched for the last decade [1,2]. Forensics techniques are based on the assumption that any post-processing of the image leaves traces in the resulting image. Therefore, a suspicious image is passively investigated without any prior knowledge (e.g., the acquisition device or source images), so that the corresponding statistical properties of manipulation are uncovered in the forensic analysis.

Within the last few decades, surveillance cameras have been installed in many areas to prevent crime. As a consequence, in many court cases, surveillance videos are used as court evidence. Unfortunately, surveillance videos can be easily forged by video editing tools, such as Premier, Vegas, *etc.* If a forged surveillance video is used as court evidence, it can cause severe problems, such as convicting an innocent person. Therefore, it is important to detect forgeries associated with surveillance videos.

**Figure 1.**
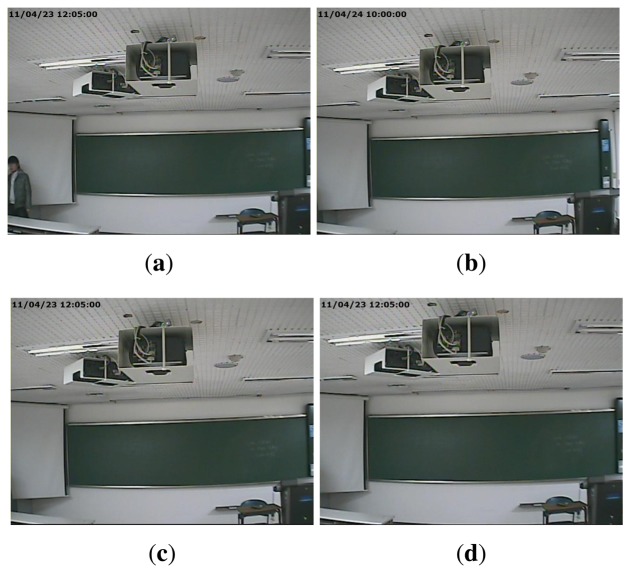
Examples of upscale-crop forgery: (**a**) a video taken at the time of a crime; (**b**) a video taken at another time; (**c**) a video forged to capture an altered time; (**d**) a video forged to alter the crime scene.

A common form of manipulation, which is often perpetrated in surveillance video, is cropping a video after enlarging it to erase evidence of a crime in the outer part of the video. As surveillance videos are always recorded at regular resolution, magnifying to the original resolution is required for a cropped video. We refer to this type of forgery as upscale-crop forgery. An upscale-crop forgery can be attempted in the following scenario. Figure 1a, b are videos taken from the same surveillance camera installed at the same place, but captured at a different time. In order to hide the crime, the offender could falsify the evidence by switching Figure 1a with Figure 1b. For this purpose, the time information of Figure 1b, which is located in the upper left corner of the video, should be manipulated. For this trick, the offender may try upscale-crop forgery in three steps. At first, the video of Figure 1b is zoomed in a little. After that, the time information is excluded by cropping outside of the zoomed video. Finally, the time information of Figure 1a is inserted into the upscale-cropped video. Figure 1c shows the result of upscale-crop forgery. Since time information is normally displayed on the outer portion of the video, the falsifier can easily manipulate a video by upscale-crop forgery. Similarly, Figure 1d can be also generated by upscale-crop forgery by erasing the offender from Figure 1a in the left corner.

**Figure 2.**
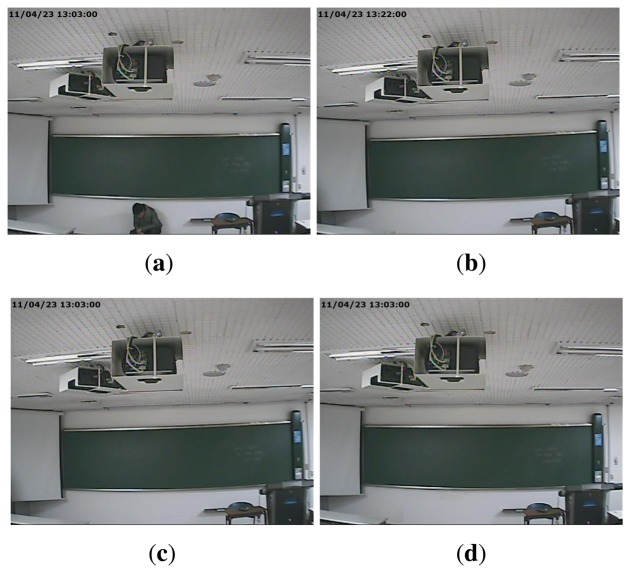
Example of partial manipulation: (**a**) a video taken at the time of a crime; (**b**) a video taken at another time; (**c**) a video forged to capture an altered time; (**d**) a video forged to alter the crime scene.

Another type of forgery, in which splicing and compositing parts of a video frame by various image editing methods, such as intra- and inter-frame copy-move, splicing, and so on, can also be accomplished. We refer to this type of forgery as partial manipulation. For instance, the falsifier can generate Figure 2c, which includes time information that was spliced from the information of Figure 2a by image editing methods. In addition, Figure 2d can be also generated by erasing the presence of the criminal from Figure 2a.

By examining the forged videos of Figures 1 and 2, we can notice that it is difficult for the naked eye to find differences between the original and forged video except for camera direction or desk arrangement, which changes every day. Thus, a forensic method is needed to analytically detect surveillance video forgery.

In this paper, we propose a forensic method for surveillance video based on sensor pattern noise (SPN). In order to detect manipulation of surveillance video with high accuracy, we first analyze the general characteristics of the surveillance camera and consider related studies from the literature in Section 2. From the analysis, an overview of SPN in Section 3 lays the foundation for its application to surveillance video forensics. Sections 4 and 5, respectively, explain the proposed upscale-crop and partial manipulation detectors based on SPN. Experimental results are then given in Section 6, and Section 7 concludes the paper.

## 2. Related Studies and Contributions

This section explains the general characteristics of surveillance video. We then review the forensic methods that have been studied so far in two separate categories: upscale-crop forgery and partial manipulation detectors. Finally, we discuss the contributions of the proposed method.

### 2.1. Characteristics of Surveillance Video

In order to detect forgeries of surveillance video, first of all, the characteristics of a surveillance camera should be analyzed. Unlike general cameras, surveillance cameras can record not only RGB video, but also infrared video. They can also take both static-scene video from a fixed position and dynamic-scene video when the camera is installed on a moving object. Furthermore, the recorded video is compressed by video codec, such as H.264. Therefore, these characteristics of surveillance cameras should be considered for a forgery detector of surveillance video.

### 2.2. Upscale-Crop Forgery Detection Methods

Enlarging a video includes a resampling process of each frame in the video. Therefore, upscale-crop forgery can be detected by determining whether or not an investigated video was resampled. Gallagher first uncovered periodicity in the second derivative signal of interpolated images [3]. Not only was the existence of the resampling process of a suspicious image estimated, but also the corresponding resampling factor of the image by analyzing the periodicity. In [4], Mahdian and Saic achieved a theoretical extension of [3]. They analyzed periodic properties that appear in the covariance structure of a resampled image; their approach was able to detect traces of geometric transformation, such as scaling, rotation and skewing. Similar work was proposed by Prasad and Ramakrishnan [5]. The authors detected resampling by analyzing a binary signal obtained by zero crossings of the second derivative of the interpolated signal, and then, they localized the portion of the image that had been tampered with.

Aside from analyzing the n-th order derivative of the interpolated image, Popescu and Farid identified the correlation between neighboring pixels of the interpolated image by a local linear predictor that uses an expectation/maximization (EM) algorithm [6]. The output of the EM algorithm, called a probability map, was then analyzed to determine if an investigated image was resampled. In [7], Kirchner analytically derived the relation between the derivative-based detector and Popescu's detector. From the analysis, he proposed an improved resampling detector. He also showed that specific periodicities can be detected in a series of tailored row and column predictors [8] instead of relying on a single predictor, as in [6].

These methods were able to detect a trace of upscale-crop forgery accurately if an investigated video was uncompressed. However, these methods rarely detect forgery in surveillance videos, because the periodic properties of resampling artifacts mostly disappear during the video compression process.

### 2.3. Partial Manipulation Detection Methods

Methods to detect partially manipulated images or videos have been actively researched in the last decade. Detection of copy-move (CM) forgery is one of representative approaches to localizing partial manipulation. CM forgery copies parts of an image and inserts them into another part of the same image. This is usually done to conceal or emphasize detail. CM forgery was first proposed by Fridrich et al. [9]. They lexicographically sorted quantized discrete cosine transform (DCT) coefficients of small blocks and, then, investigated whether the adjusted blocks were similar or not. Wang and Farid proposed a method for detecting CM forgery of a video by correlation between the original regions and cloned ones [10]. They first examined the existence of duplicated frames among video frames and then examined duplicated regions of each frame. Their method showed appropriate performance for both dynamic- and static-scene video. Until now, in addition to the above methods, many CM forgery detectors have been proposed [11–15]. Even though these methods accurately detect partial manipulation in a variety of environments, they cannot identify if forged regions are copied from other images or video segments.

To overcome the drawbacks of CM forgery detectors, methods that analyze the statistical properties of both unforged and forged areas have been proposed. Chen et al. proposed the detection method of partial manipulation based on SPN [16]. They revealed doctored regions by analyzing the presence of SPN in the investigated image. However, there was less accuracy when extracted SPN contained many high-frequency components in the scene. Mondaini et al. presented a method for the detection of forgeries in digital video using SPN [17]. They calculated and exploited three correlations, C1 (correlation between the reference SPN and the SPN from a frame of test video), C2 (correlation between frames) and C3 (correlation between the SPN extracted from two consecutive frames). However, their method only used C1 for revealing doctored regions within a frame. Thus, for detecting partial manipulation, Mondaini *et al.*'s approach is the same as the approach of Chen *et al.* [16]. Therefore, Mondaini *et al.*'s method also had low accuracy when the SPN contained many high-frequency components. Hsu *et al.* developed a method based on the correlations of SPN between every two temporally neighboring video blocks [18]. Simulation results showed that the temporal correlation was a fairly reliable feature in fine-quality video, but it was sensitive to video compression. In addition, if reference SPN can be cleanly estimated, their method had lower accuracy than an approach based on correlation between reference SPN and test SPN, such as in [16,17]. Kobayashi et al. developed a method to detect doctored regions in static-scene video based on the noise level function (NLF) [19]. They estimated the NLF of video at each pixel to detect manipulation. In contrast to [16], Kobayashi's method could not be applied to dynamic-scene video. Moreover, their method was less accurate if the test video was compressed. Conotter *et al.* described a geometric method to detect physically implausible trajectories of objects in video sequences [20]. They explicitly modeled the three-dimensional projection of the trajectory into the image plane of a static or moving camera. Their method was largely insensitive to resolution, post-processing, compression and re-compression. However, only forged objects, which are physically moved by gravity, can be detected. Thus, it is difficult to apply that method to surveillance video, which is generally manipulated in the region of criminal or time information.

### 2.4. Main Contributions

In our previous work, we proposed a forgery detection method that could detect upscale-crop forgery and partial manipulation [21]. Upscale-crop forgery was detected by using a resampling estimation method [3] and SPN, and partial manipulation was found by analyzing SPN in a local searching window; this method was similar to the method in [16]. However, our previous method also had some of the drawbacks mentioned in Section 2.2 and 2.3, such as low accuracy in static-scene and compressed video. To address these limitations, we propose an improved method that considers the characteristics of the surveillance camera, such as RGB/infrared mode, dynamic-/static-scene and video compression.

We discuss three main contributions to solving the problems of surveillance camera forensics. To satisfy the inherent constraints of a surveillance camera as stated in Section 2.1, SPN is used as a statistical tool. SPN is extractable from both the RGB video and the infrared video. Moreover, the SPN survives under lossy compression, filtering, gamma correction and many other typical processing procedures. Therefore, the proposed method is robust against RGB/infrared mode and video compression (see Section 3 for the details of SPN). A second contribution lies in a novel upscale-crop forgery detector based on a minimum average correlation energy Mellin radial harmonic (MACE-MRH) correlation filter. To detect upscale-crop forgery, we designed two different MACE-MRH correlation filters from a reference SPN (see Section 4). Third, we propose the partial manipulation detector to accurately detect partial manipulation in static-scene video by eliminating high frequency components and adaptively choosing the size of a local search window (see Section 5).

## 3. Sensor Pattern Noise

An image sensor, such as CCD or CMOS, in digital imaging devices is composed of an enormous number of photon detectors, commonly called pixels. Pixels have different sensitivities to light, due to the inhomogeneity of silicon wafers, and the variable sensitivity of each pixel to light generates uncorrelated pattern noise. Consequently, every sensor casts a unique sensor pattern noise (SPN) onto every image or video it takes. This SPN acts as a sensor fingerprint and exploits identifying imaging sources or verifying the image in the field of digital forensics [22–24].

In a video clip, the SPN can be extracted as follows. Assuming that the video is composed of *N* frames, *I*_1_, *I*_2_, …, *I_n_*, the SPN of the video is computed as Equation (1) [23].

(1) $$SPN=\overset{N}{\underset{k=1}{\text{∑}}}\frac{W_{k}{\hat{I}}_{k}}{{\left({\hat{I}}_{k}\right)}^{2}}\text{where}\;{\hat{I}}_{k}=F\left(I_{k}\right),W_{k}=I_{k}-{\hat{I}}_{k}$$

where *F* is the wavelet denoising filter described in [24].

**Figure 3.**
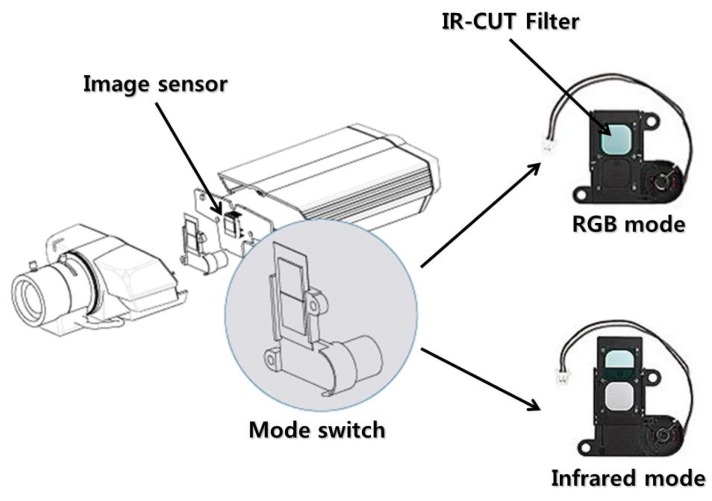
The structure of surveillance video recording both RGB and infrared video.

Using SPN extracted from Equation (1), we aimed to detect forgeries of surveillance video. SPN is suitable for detecting surveillance video forgery, due to two characteristics. First, SPN extracted from the infrared mode is the same as that of the RGB mode. As seen in Figure 3, a surveillance camera records RGB video and infrared video using a mode switch that automatically opens and closes the IR-CUT filter. As the IR-CUT filter only affects the wavelength of light coming into the image sensor, SPN, generated by the error of the sensitivity of light, is not affected by the mode switch. Thus, the same SPN appears on the RGB and infrared video if they are taken by the same camera. Second, SPN is robust against video compression [25]. Since SPN is evenly distributed across every pixel of an investigated video, much of the information on SPN survives even after harsh compression.

**Figure 4.**
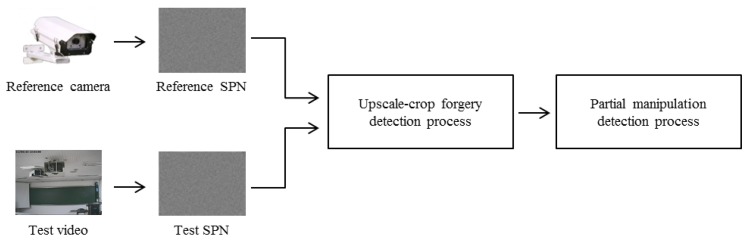
The whole process of the proposed method.

The whole process of the proposed method, which is based on the characteristics of SPN, is depicted in Figure 4. After extracting the reference and test SPN from a reference camera and a test video, respectively, we first investigate a trace of upscale-crop forgery. Afterwards, the existence of partial manipulation is inspected. Each process of manipulation detection is described in Sections 4 and 5.

## 4. Upscale-Crop Forgery Detection Process

This section explains the details of upscale-crop forgery detection as the first step of the proposed method. In general, when a surveillance video is presented as legal evidence in court, the information of the camera, by which the video is recorded, becomes available publicly. Thus, prior knowledge of the recording device can be used to determine whether or not the investigated video is manipulated.

To detect upscale-crop forgery of the surveillance video, we exploit reference SPN extracted from the source camera. More specifically, two MACE-MRH correlation filters, which have different scale-tolerance ranges, are designed from the corresponding reference SPN. The first filter is designed to have a very large correlation value when the suspicious video is not scaled. On the other hand, the second filter is designed to maintain a high correlation value even if the suspicious video is upscaled. By calculating two cross-correlation values between the different MACE-MRH correlation filters and the test SPN, we judge whether or not the test video is forged.

In the following subsections, we first provide an overview of the MACE-MRH correlation filter [26]. Afterwards, we describe how the MACE-MRH correlation filter is applied to the proposed forgery detector.

### 4.1. MACE-MRH Correlation Filter

Correlation is a natural metric for characterizing the similarity between a reference pattern and a test pattern. A correlation filter is designed to have a high correlation peak if the reference pattern, which was used to generate the filter, is similar to a test pattern. Let *x*(*m*, *n*) and *h*(*m*, *n*) denote the 2D test pattern and the correlation filter generated from the reference pattern, respectively. Then, correlation output *c*(*m*, *n*) can be expressed as the inverse 2D Fourier Transform (FT) of the conjugate product in the frequency domain as follows:

(2) $$c(m,n)=FT^{-1}\{X(k,l)H^{*}(k,l)\}$$

where *X*(*k*, *l*) and *H*(*k*, *l*) are the 2D FT of *x*(*m*, *n*) and *h*(*m*, *n*), respectively. If the test pattern is similar to the reference pattern, the correlation output, *c*(*m*, *n*), has a high correlation peak. Thus, the similarity betweien two patterns is measured by calculating the following peak to correlation energy (PCE).

(3) $$PCE=\frac{c{\left(i_{\text{peak}},j_{\text{peak}}\right)}^{2}}{\sum_{i,j}c{\left(i,j\right)}^{2}}$$

The correlation filter basically has a shift invariance property, since the convolution operation in Equation (2) effectively projects a test pattern onto the filter pattern regardless of shifting [27]. Due to the shift invariance property, the correlation filter ensures a high correlation value even after shifting or cropping of the test pattern. Furthermore, beyond the basic correlation filter, improved correlation filters have been studied (e.g., achieving sharpness, noise-tolerance or distortion-tolerance while preserving the shift invariance property) [26–30]. Among many filters, the minimum average correlation energy Mellin radial harmonic (MACE-MRH) correlation filter achieves scale-tolerance in the allowed range of scale distortion [26].

**Figure 5.**
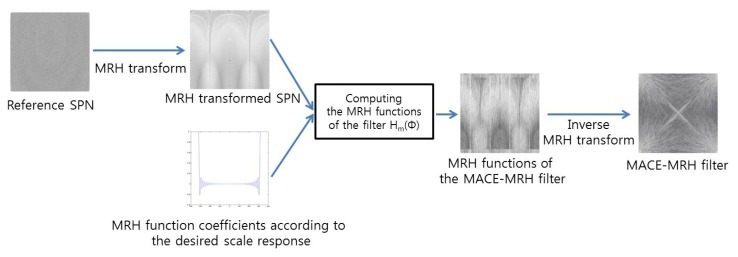
Design procedure of a minimum average correlation energy Mellin radial harmonic (MACE-MRH) correlation filter.

To detect upscale-crop forgery, the proposed method exploits the MACE-MRH correlation filter designed from reference SPN. The MACE-MRH filter is designed as shown in Figure 5. At first, the Mellian radial harmonic (MRH) function, *F_m_*(*ϕ*), is computed from reference SPN:

(4) $$\begin{array}{l} F_{m}(\phi )=L^{-1}\int_{r_{0}}^{R}F\left(\rho ,\phi \right)\rho^{-j2\pi m-1}\rho d\rho \\ F\left(\rho ,\phi \right)=\overset{\infty }{\underset{m=-\infty }{\text{∑}}}F_{m}\left(\phi \right)\rho^{j2\pi m-1} \end{array}$$

where *F*(*ρ,ϕ*) is the FT of the reference SPN in polar coordinates and *F_m_*(*ϕ*) is the m-th MRH function of *F*(*ρ*, *ϕ*). *R* and *r*_0_ are selected as 768 and 14.07, respectively, which satisfy the relationship ln *R* – ln r_0_ = *L* ∈ Z^+^.

Second, MRH function coefficients are computed according to the scale response. The relationship between the scale response, *c*(*β*), and the coefficients, *C_m_*, represents the function of the scale factor, *β*, as below:

(5) $$c\left(\beta \right)=\beta^{-1}\overset{\infty }{\underset{m=-\infty }{\text{∑}}}e^{-j2\pi \ln\beta }C_{m}\;\text{where}\;C_{m}=\int_{0}^{2\pi }F_{m}\left(\phi \right)H_{m}^{*}\left(\phi \right)d\phi$$

where *H_m_*(*ϕ*) is the m-th MRH function of the MACE-MRH correlation filter. Equation (5) can be simplified by an invertible logarithmic transformation, Λ, so that we yield *ĉ*(*β*) in Equation (6)

(6) $$\hat{c}\left(\beta \right)=\wedge \{c\left(\beta \right)\}=e^{\frac{L\beta }{2\pi }}\cdot c(e^{\frac{L\beta }{2\pi }})=\overset{\infty }{\underset{m=-\infty }{\text{∑}}}C_{m}e^{-j(Lm)\beta }$$

The relationship between the transformed scale response, *ĉ*(*β*), and the coefficients, *C_m_*, is exactly the same as *A*(*w*) and *a_k_* of Equation (7), which represent the frequency response of the finite impulse response (FIR) filter and the k-th coefficient of the impulse response, respectively.

(7) $$A(w)=\overset{M}{\underset{k=-M}{\text{∑}}}a_{k}e^{-jkw}$$

The similarity of Equations (6) and (7) can make the problem of calculating MRH functions coefficients *C_m_* as that of designing the FIR filter of *A*(*w*). Thus, by employing available FIR filter design algorithms, the coefficients, *C_m_*, of *c*(*β*) can be calculated. In this paper, we calculated *C_m_* by the fir1 function of Matlab 2012a.

After that, the MRH function, *H_m_*(*ϕ*), of Equation (5) is determined by using *C_m_* as constraints. There are many *H_m_*(*ϕ*) candidates that satisfy the constraints. Thus, more constraints are needed to find a unique solution. Therefore, we selected *H_m_*(*ϕ*), which minimize the average correlation energy while satisfying the relationship in Equation (5) as below:

(8) $$\begin{array}{l} \text{Find}H_{m}(\phi )\text{to minimize}\\ \;\int_{0}^{2\pi }{\left|H_{m}(\phi )\right|}^{2}P_{F}(\phi )d\phi \\ \text{subject to}\\ \;\int_{0}^{2\pi }F_{m}(\phi )H_{m}^{*}(\phi )d\phi =C_{m} \end{array}$$

where *P_f_*(*ϕ*) is the average power spectrum of the reference SPN obtained by computing the average of *F*(*ρ*, *ϕ*)*^2^* along the radial axis. Equation (8) can be solved by Lagrange multipliers, and the solution to the minimization problem of Equation (8) is:

(9) $$\begin{array}{ll} H_{m}(\phi )=\lambda_{m}^{*}\frac{F_{m}(\phi )}{P_{F}(\phi )+\alpha }, & \lambda_{m}=\frac{C_{m}}{\int_{0}^{2\pi }\frac{{\left|F_{m}(\phi )\right|}^{2}}{P_{F}(\phi )+\alpha }d\phi }. \end{array}$$

where α is a small additive constant. We calculated *H_m_*(*ϕ*) from *C_m_* and *F_m_*(*ϕ*) by Equation (9).

Finally, the correlation filter, *H*(*u,v*), is found by applying the inverse MRH transforms of *H_m_*(*ϕ)* and applying a polar-to-Cartesian coordinate transform.

### 4.2. Upscale-Crop Forgery Detection Using the MACE-MRH Correlation Filter

In the previous section, we explained how the MACE-MRH correlation filter is designed. In this section, we show how we apply the MACE-MRH correlation filter to upscale-crop forgery detection.

**Figure 6.**
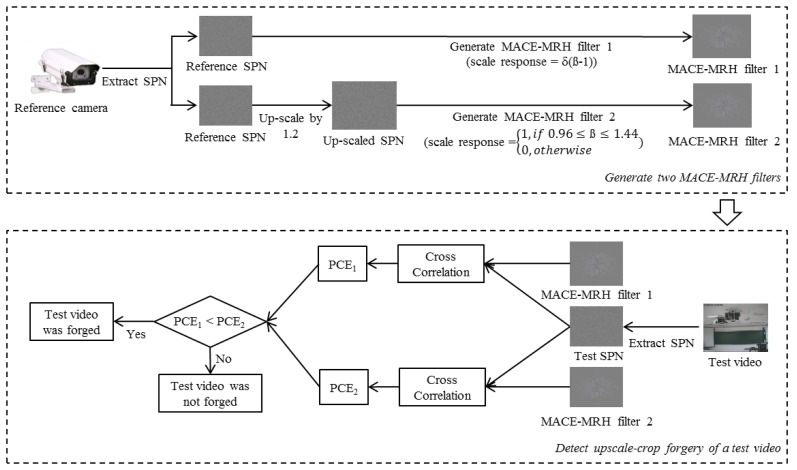
Proposed upscale-crop forgery detection procedure.

In our previous work, we proposed a camera identification method using a MACE-MRH correlation filter generated from SPN [31]. The experiments from that work proved that the MACE-MRH correlation filter can be applied to SPN, which has very weak energy, because the MACE-MRH filter can control the intensity of distortion, and that did not distort the test SPN, but only the reference SPN. From these facts, we apply the MACE-MRH correlation filter, which was generated from the reference SPN, to the upscale-crop forgery detector. The whole process is depicted in Figure 6. At first, a reference video was generated by a surveillance camera that was used to record the test video. The reference SPN was extracted from the reference video. After that, two MACE-MRH correlation filters were designed from reference SPN. The first correlation filter was designed to have a peak value in the correlation output when the test pattern was not scaled. If *C_m_* = 1 for all *m*, the scale response of Equation (5) is simplified as follows:

(10) c(β)=δ(β−1)

Equation (10) shows that the correlation output has a peak only if *β* = 1. Therefore, by designing the first MACE-MRH correlation filter with *C_m_* = 1 for all *m*, we can generate a filter that has only a peak correlation value when the input pattern is not distorted by scaling.

The second MACE-MRH correlation filter is designed to maintain a high correlation value even if the input pattern is enlarged. To design such a filter, the reference SPN is magnified by *T* times. Then, the MACE-MRH correlation filter is generated from the magnified reference SPN and the scale response, *c*(*β*), is set as below.

(11) $$c(\beta )=\{\begin{array}{ll} 1, & if\;1-\beta_{t}\leq \beta \leq 1+\beta_{t}\\ 0, & \text{otherwise} \end{array}$$

As the filter is generated from the reference SPN magnified by *T* times, it tolerates scaling in the following range.

(12) $$T\times (1-\beta_{t})\leq \text{scaling factor}\leq T\times (1+\beta_{t})$$

**Figure 7.**
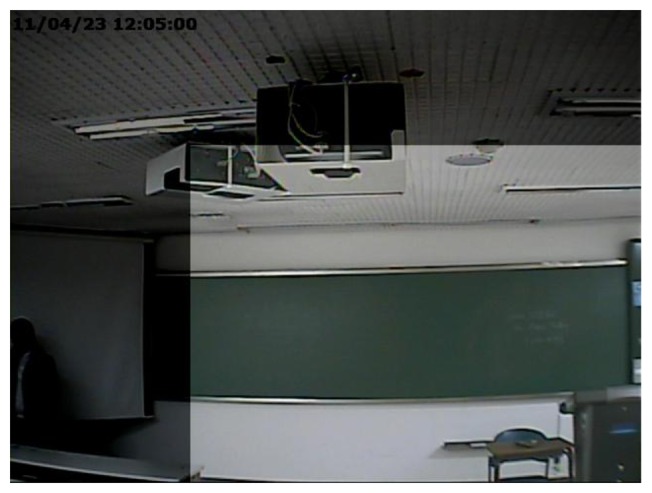
A cropped region after scaling by 1.4.

Because of the trade-off between the range of scale-tolerance and the performance, the MACE-MRH filter generally has a higher correlation value with a lower *β_t_* value [26]. Thus, we should properly choose *β_t_* to detect forgery in the surveillance video compressed with low quality. In a real world scenario, attackers may magnify the video with a small scaling factor, because they do not want to expose their manipulation. Thus, it is unnecessary to consider a wide range of scaling factors that can be easily detected by the naked eye. Figure 7 shows a cropped area when an upscale-crop is attempted with a scaling factor of 1.4. Since about 49% of the video is cropped out, the forgery would be easily detected, even by the naked eye. Thus, we assume that the inspected video is upscaled with a scaling factor of less than 1.4 and, then, cropped to original size. With these constraints, the proposed method designs the second MACE-MRH correlation filter tolerant in a range of 0.96 ≤ *scaling factor* ≤ 1.44 by setting *T* = 1.2 and *β_t_* = 0.2 in Equation (12).

After designing two filters, namely *χ*_1_ and *χ*_2_, we extract test SPN *ψ* from the inspected video by Equation (1). Then, cross-correlation between them is computed as follows.

(13) $$\begin{array}{c} c_{1}(x,y)=\overset{M}{\underset{m_{1}=0}{\text{∑}}}\overset{N}{\underset{n_{1}=0}{\text{∑}}}\chi_{1}(m_{1},n_{1}).\psi (m_{1}+x,n_{1}+y)\\ c_{2}(x,y)=\overset{M}{\underset{m_{2}=0}{\text{∑}}}\overset{N}{\underset{n_{2}=0}{\text{∑}}}\chi_{2}(m_{2},n_{2}).\psi (m_{2}+x,n_{2}+y) \end{array}$$

Finally, PCE values are calculated from the two correlation outputs to judge whether or not the test video was forged.

(14) $$\begin{array}{c} PCE_{1}=\frac{C_{1}{\left(m_{1_{\text{peak}}},n_{1_{\text{peak}}}\right)}^{2}}{\frac{1}{MN}\overset{M}{\underset{m_{1}=0}{\text{∑}}}\overset{N}{\underset{n_{1}=0}{\text{∑}}}C_{1}{\left(m_{1},n_{1}\right)}^{2}}\\ PCE_{2}=\frac{C_{2}{\left(m_{2_{\text{peak}}},n_{2_{\text{peak}}}\right)}^{2}}{\frac{1}{MN}\overset{M}{\underset{m_{2}=0}{\text{∑}}}\overset{N}{\underset{n_{2}=0}{\text{∑}}}C_{2}{\left(m_{2},n_{2}\right)}^{2}} \end{array}$$

where (*m*_1_*_peak_*,*n*_1_*_peak_*) and (*m_2peak_*,*n_2peak_*) are the peak positions in *c*_1_ and *c*_2_, respectively, which represent the location of the maximum values from *c*_1_ and *c*_2_.

**Figure 8.**
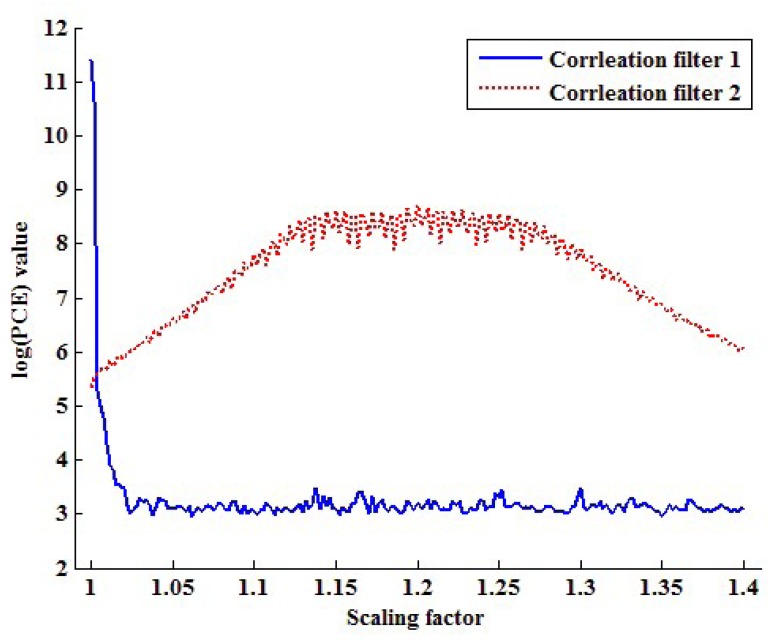
Plots for the peak to correlation energy (PCE) values of the two correlation filters varying the scaling factor.

Figure 8 plots the PCE values of each filter by changing the scaling factor from one to 1.4. As seen in Figure 8, the PCE value of the first correlation filter is larger than that of the second correlation filter if the scaling factor is one. On the other hand, the correlation value of the second correlation filter is larger than that of the first correlation filter in other scaling factors. Based on this fact, the proposed method determines that the inspected video is not forged if the *PCE*_1_ value is larger than the *PCE_2_* value and *vice versa*.

## 5. Partial Manipulation Detection Process

**Figure 9.**
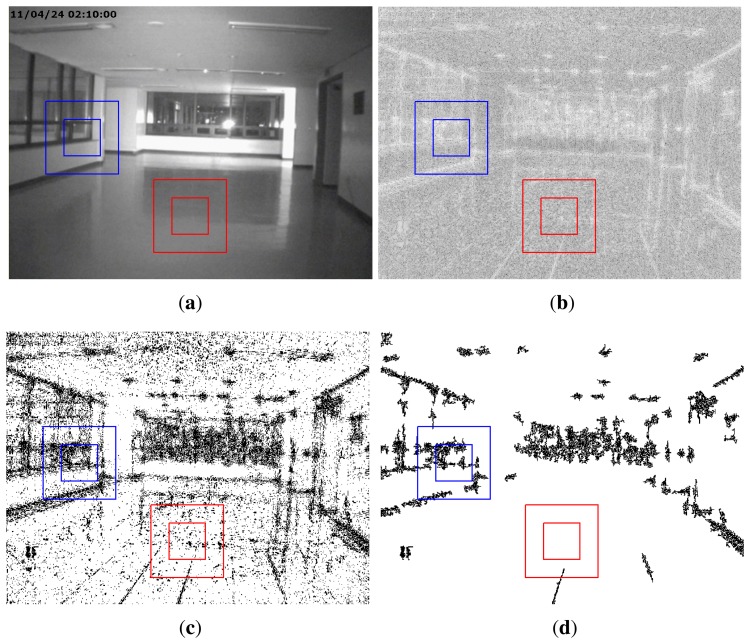
(**a**) A non-forged static-scene video; (**b**) sensor pattern noise (SPN) extracted from (**a**); (**c**) estimated high-frequency map from (**b**); (**d**) revised high-frequency map.

In this section, we explain how to detect partial manipulation of a video under investigation. If a video has been partially manipulated by clipping objects from an intra-or inter-frame and superimposing them, the doctored region will have weak SPN or not be synchronized with the reference SPN. Thus, the normalized correlation coefficient (NCC) between the reference and test SPN in the doctored region is very small. By using this property of SPN, Chen *et al.* detected doctored regions in an image [16]. However, Chen's approach had the drawback of having low accuracy when extracted SPN contained many high-frequency components of an image or a video. Figure 9a,b shows an unforged static-scene video and the corresponding SPN, respectively. As shown in Figure 9b, we can notice that part of the SPN is contaminated by high-frequency components of the video. These high-frequency components are not SPN, but the scene information of the video. Thus, the NCC of the region that contains many high-frequency components would be small, although the region has not been tampered with. Therefore, detecting forgery in a static-scene video needs an approach that excludes high-frequency components from SPN.

Li proposed an approach to reduce the influence of high frequency components in their SPN [32]. By applying Li's enhanced SPN to Chen's method, the forgery of static-scene video can be more accurately detected. However, the accuracy of that approach decreases if the size of the local searching window is small. Because the resolution of a surveillance camera is generally lower than that of a general camera, the size of a manipulated region would be relatively small. Thus, for accurately detecting forgery in a surveillance video, the size of the local searching window should be as small as possible.

From the above observation, we propose a forgery detector with a small local searching window to accurately detect partial manipulation from a static-scene video. Specifically, the proposed method estimates a high frequency map (HFM) to determine whether or not each pixel of the SPN is contaminated by high frequency components. Afterwards, we remove the high frequency components from the SPN using the HFM and adaptively select the size of the local searching window according to the quantity of clean SPN in the window block. In the next subsection, we explain the details of estimating the HFM and forgery detection method.

### 5.1. Estimating a High Frequency Map

SPN is accurately estimated with smooth and bright scenes, such as a scene of blue sky [22]. Thus, by modeling clean SPN distribution from SPN values of a blue sky video and using that distribution, we can effectively exclude a contaminated part of SPN from an investigated SPN. More specifically, we captured blue-sky videos with a resolution of 640 × 480 from ten cameras in Table 1 and, then, extracted 3,072,000 pixel values from the SPN values of the videos. Afterwards, we modeled the distribution of clean SPN by fitting these pixels with the following Gaussian function.

(15) $$G(x)=\alpha \cdot \exp(-{\left((x-\mu )/\sigma \right)}^{2})$$

where *α* = 1.703*e* + 0.005, *μ* = 0, and *σ* = 0.000944.

**Figure 10.**
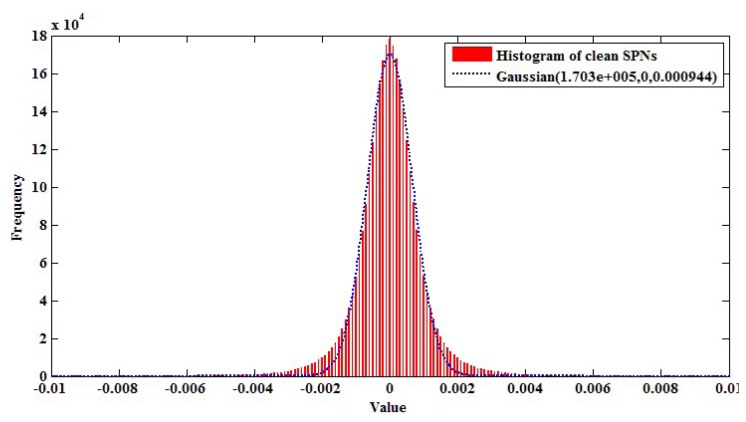
Histogram of clean SPN.

Figure 10 shows the modeled clean SPN distribution. By using that distribution, we determine SPN values, whose probability is larger than 10^−5^ with threshold *τ_spn_*, as high frequency components. In other words, a high frequency map (HFM) is determined from the investigated SPN as in the following equation.

(16) $$HFM(x,y)=\{\begin{array}{cc} 0, & \left|\psi (x,y)\right|>\tau_{spn}\\ 1, & \left|\psi (x,y)\right|\leq \tau_{spn} \end{array}$$

where *ψ* is test SPN, *τ_spn_* is 0.003 and *HFM* indicates whether or not the corresponding SPN pixel is a high frequency component (0 = high frequency component, 1 = clean SPN).

After estimating the *HFM*, we prune the erroneously detected part of the *HFM*. Since high frequency components are normally connected with their neighbors, connected *HFM* components, whose size is smaller than 10 × 10, are filtered out using the four-connected-component labeling algorithm [33]. Figure 9d shows the corrected *HFM* of Figure 9c. As shown in Figure 9d, the proposed method accurately separates the contaminated SPN from the investigated SPN.

Using the *HFM* calculated as above, the proposed method removes the parts of contaminated SPN by multiplying the HFM with the corresponding SPN and calculates the NCC from the filtered SPN. Table 2 depicts the NCC values calculated from the four blocks of Figure 9b using Chen's method [16], Li's method [32] and the proposed *HFM*. The size of the small block is 64 × 64, and that of the large block is 128 × 128. NCC values between each block of test SPN and the corresponding reference SPN were calculated as below.

(17) $$NCC=\frac{\sum_{x,y}\left(\chi (x,y\right)-\bar{\chi }\text{)}\cdot \text{(}\psi (x,y)-\bar{\psi })}{\sqrt{{\sum_{x,y}\left(\chi (x,y)-\bar{\chi }\right)}^{2}\cdot \sum_{x,y}{\left(\psi (x,y)-\bar{\psi }\right)}^{2}}}$$

where *ψ* and *χ* represent the selected block of test SPN and the corresponding reference SPN, respectively. The test SPN of the proposed *HFM* and Li's method was filtered by Equation (18) and Model 5 of Li's method, respectively

**Figure 11.**
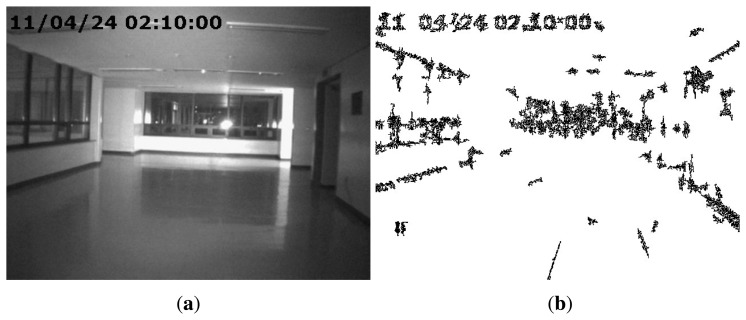
(**a**) A non-forged video with a big time stamp; (**b**) the high frequency map (HFM) estimated from (**a**).

The position of the time stamp in the video does not contain any sensor pattern noise, as the time stamp was digitally inserted after the capture. Thus, if a test video has a big time stamp, SPN estimated from that video will have many errors in the region of the time stamp. Therefore, the SPN errors by the time stamp should be filtered. The proposed method can filter them by *HFM*, which determines that the position of the time stamp is not SPN, as shown in Figure 11.

### 5.2. Detection of Partial Manipulation Based on the HFM

The proposed method detects partial manipulation in static-scene video by eliminating high frequency components using the *HFM* and adaptively choosing the size of the local search window according to the quantity of clean SPN in the window block. The detection process of partial manipulation consists of three steps. In the first step, we generate clean SPN for the test video by removing contaminated SPN. To that purpose, the *HFM* estimated from the test SPN is multiplied by both the reference and test SPN.

(18) $$\begin{array}{c} \hat{\delta }=\delta \cdot HFM\\ \hat{\psi }=\psi \cdot HFM \end{array}$$

where *δ* and *ψ* represent the reference and test SPN, respectively.

In the second step, to determine whether or not any pixel of the test video is forged, NCC values between *δ̂* and *ψ̂* are calculated by sliding a searching window centered at each pixel. The size of the local searching window is adaptively determined by the quantity of clean SPN in the window block, as shown in Equation (19).

(19) $$\begin{array}{l} BS(x,y)=\underset{\alpha \in \left[64,128\right]}{\arg\min}\overset{\alpha /2-1}{\underset{i=-\alpha /2}{\text{∑}}}\overset{\alpha /2-1}{\underset{j=-\alpha /2}{\text{∑}}}HFM(x+i,y+j)\\ \text{with the constraint}\\ \overset{\alpha /2}{\underset{i=-\alpha /2}{\text{∑}}}\overset{\alpha /2}{\underset{j=-\alpha /2}{\text{∑}}}HFM(x+i,y+j)\geq 64\times 64 \end{array}$$

where *BS*(*x, y*) is the block size of the window centered on (*x, y*). Once the block size of the window is determined, each inspection block is extracted from *δ̂* and *ψ̂* as follows:

(20) $$\begin{array}{l} \delta_{\text{block}(x,y)}=\left[\begin{array}{llll} {\hat{\delta }}_{\left(x-T,y-T\right)} & {\hat{\delta }}_{\left(x-T+1,y-T\right)} & \cdots & {\hat{\delta }}_{\left(x+T-1,y-T\right)}\\ {\hat{\delta }}_{\left(x-T,y-T+1\right)} & {\hat{\delta }}_{\left(x-T+1,y-T+1\right)} & \cdots & {\hat{\delta }}_{\left(x+T-1,y-T\right)}\\ \vdots & \vdots & & \vdots \\ {\hat{\delta }}_{\left(x-T,y+T-1\right)} & {\hat{\delta }}_{\left(x-T+1,y+T-1\right)} & \cdots & {\hat{\delta }}_{\left(x+T-1,y+T-1\right)} \end{array}\right]\\ \psi_{\text{block}(x,y)}=\left[\begin{array}{llll} {\hat{\psi }}_{\left(x-T,y-T\right)} & {\hat{\psi }}_{\left(x-T+1,y-T\right)} & \cdots & {\hat{\psi }}_{\left(x+T-1,y-T\right)}\\ {\hat{\psi }}_{\left(x-T,y-T+1\right)} & {\hat{\psi }}_{\left(x-T+1,y-T+1\right)} & \cdots & {\hat{\psi }}_{\left(x+T-1,y-T\right)}\\ \vdots & \vdots & & \vdots \\ {\hat{\psi }}_{\left(x-T,y+T-1\right)} & {\hat{\psi }}_{\left(x-T+1,y+T-1\right)} & \cdots & {\hat{\psi }}_{\left(x+T-1,y+T-1\right)} \end{array}\right] \end{array}$$

where *T* is *BS*(*x, y*)/2, and NCC values between *δ*_*block*(*x,y*)_ and *ψ*_*block*(*x,y*)_ are calculated.

(21) $$\rho (x,y)=\frac{\sum_{i,j}\left[\delta_{\text{block}(x,y)}(i,j)-\bar{\delta_{\text{block}(x,y)}}\right]\;\left[\psi_{\text{block}(x,y)}(i-x,j-y)-\bar{\psi_{\text{block}(x,y)}}\right]}{{\left\{{\sum_{i,j}\left[\delta_{\text{block}(x,y)}(i,j)-\bar{\delta_{\text{block}(x,y)}}\right]}^{2}\sum_{i,j}{\left[\psi_{\text{block}(x,y)}(i-x,j-y)-\bar{\psi_{\text{block}(x,y)}}\right]}^{2}\right\}}^{0.5}}$$

If the inspected video has been partially tampered with, the correlation between the block of reference and test SPN will be small in the doctored region. Therefore, we determine the pixel, (*x,y*), is forged if *ρ*(*x,y*) ≤ *τ_pixel_*.

(22) $$Z(x,y)=\{\begin{array}{cc} 0, & \rho (x,y)>\tau_{\text{pixel}}\\ 1, & \rho (x,y)\leq \tau_{\text{pixel}} \end{array}$$

where *Z*(*x, y*) indicates whether each pixel is forged (one) or not (zero).

As a final step, the proposed method determines whether or not the inspected video is forged as follows:

(23) $$\begin{array}{ll} \text{Video was forged} & :D>\tau_{\text{video}}\\ \text{Video was not forged} & :D\leq \tau_{\text{video}} \end{array}\text{where}D=\sum_{x,y}Z(x,y)$$

The proposed method determines that the inspected video was partially manipulated if the number of forged pixels are larger than τ*_video_* and *vice versa*.

## 6. Experimental Results

This section contains extensive detection tests of both upscale-crop forgery and partial manipulation. We recorded 120 sample videos, 60 RGB and 60 infrared videos, from ten surveillance cameras, as summarized in Table 1. Half of the sample videos were recorded as static-scene video by fixed surveillance cameras, and the other half were recorded as dynamic-scene video by moving cameras. All the sample videos were taken with a resolution of 640 × 480, a frame rate of 30 Hz and 30 s recoding time. The sample videos were compressed by an ffdshow H.264 encoder (one pass quality mode) with a quality factor of 100 [34]. Other conditions, such as white balance, sharpness, contrast, *etc.*, were automatically set.

## 6.1. Upscale-Crop Forgery Detection Experiments

In order to measure the performance of the proposed upscale-crop detector, we first created 240 unforged videos by compressing 120 sample videos with the H.264 encoder with quality factors of 80 and 100. Then, 1,920 forged videos were created as follows: All frames of 120 sample videos were enlarged in increments of 0.05 from 1.05 to 1.4 by bi-cubic interpolation kernels. The outer part of the scaled videos was then cropped out to make the resolution 640 × 480. Finally, the forged videos were compressed with quality factors of 80 and 100. With the test videos, we measured the average detection accuracy of the proposed method, varying both scaling and quality factors. We further benchmarked two more detectors, which were, respectively, proposed by Mahdian [4] and Kirchner [8]. Since their methods focused on still images, we computed the output of their methods from each frame and, then, averaged them.

The proposed method found non-doctored videos with 100% accuracy, i.e., without any false positive error. For measuring the forgery detection accuracy with a similar false positive condition, the decision thresholds of Mahdian's approach and Kirchner's approach were set to a false positive rate (FPR) of 10^−2^. After that, the forgery detection accuracy of each method was computed. Table 3 depicts the detection accuracy of three detectors varying both scaling and quality factors. The proposed method detected almost every forgery with an accuracy of over 98%. On the other hand, Mahdian's method and Kirchner's method had an average accuracy of 73.5% and 54.5%, respectively, for the compressed videos with a quality factor of 80. Further, their methods revealed low detection accuracy for small scaling factors (e.g., 1.05 and 1.1).

### 6.2. Detection Experiments for Partial Manipulation after Upscale-Crop

In this section, we discuss the interesting case in which a video is forged by both upscale-crop and partial manipulation. If upscale-crop forgery occurs after partial manipulation, the proposed detector is not affected by partial manipulation at all, since all pixels in the investigated video frame have the trace of upscaling inherently. On the other hand, if partial manipulation occurs after upscale-crop forgery, some of the upscale traces in the partially manipulated region disappear. Consequently, the detection accuracy of upscale-crop forgery might be affected by the partial manipulation after the upscale-crop, especially when the size of the doctored region is big enough.

To measure the performance of the proposed upscale-crop detector according to the size of the locally doctored region after upscale-crop forgery, 1,920 forged videos were created as follows. One-hundred twenty sample videos were enlarged with scaling factors of 1.15 and 1.3, followed by cropping to 640 × 480 at the center position. After that, the videos were forged by copying and pasting objects from other videos at random positions. The size of the doctored regions was varied from 50 × 50 to 200 × 200 in steps of 50 square pixels. Afterwards, the videos were compressed by an ffdshow H.264 encoder with quality factors of 80 and 100. With the forged videos, we measured the average detection accuracy of the proposed method. We also tested both the Mahdian [4] and Kirchner [8] detectors for comparison. The decision thresholds of their detectors were set as explained in Section 6.1

Table 4 shows the average detection accuracy of each method. The accuracy of Mahdian's method is significantly decreased in the partial manipulated video with a 200 × 200 block, while Kirchner's method is weakly affected by the size of the doctored region. However, Kirchner's method has very low accuracy, with an average of 53.2% in the compressed video with a quality factor of 80. On the other hand, the proposed method detects 98.3% of forgeries on average in all the test videos. The experimental results show that the proposed upscale-crop detector is robust against partial manipulation and video compression.

### 6.3. Partial Manipulation Detection Experiments at the Pixel Level

Sections 6.3 and 6.4 only consider the detection performance of the proposed method against forged videos with partial manipulation. In this section, we focus on how well the proposed method detects forged pixels in the locally doctored videos by evaluating the partial manipulation detection performance at the pixel level. For this purpose, 1,440 test videos were created as follows: 120 sample videos were forged by copying and pasting objects of other videos at random positions. The size of the doctored regions was varied from 100 × 100 to 150 × 150 in steps of 10 square pixels to examine the influence of the size of the doctored region. Afterwards, the videos were compressed by an ffdshow H.264 encoder with quality factors of 80 and 100. To analyze the performance according to the types of video scene, we classified the test videos into static-scene and dynamic-scene video.

To evaluate the performance of the proposed partial manipulation detector, we conducted three types of experiments. First, we analyzed the distribution of the NCC values in doctored and non-doctored regions. The NCC values corresponding to each pixel of video were calculated by Equation (21) in all the test videos. Furthermore, we benchmarked Chen's method [16] and Li's method [32]. To evaluate their methods, we selected two sizes (64 × 64 and 128 × 128) of local searching windows and computed the corresponding NCC values.

**Figure 12.**
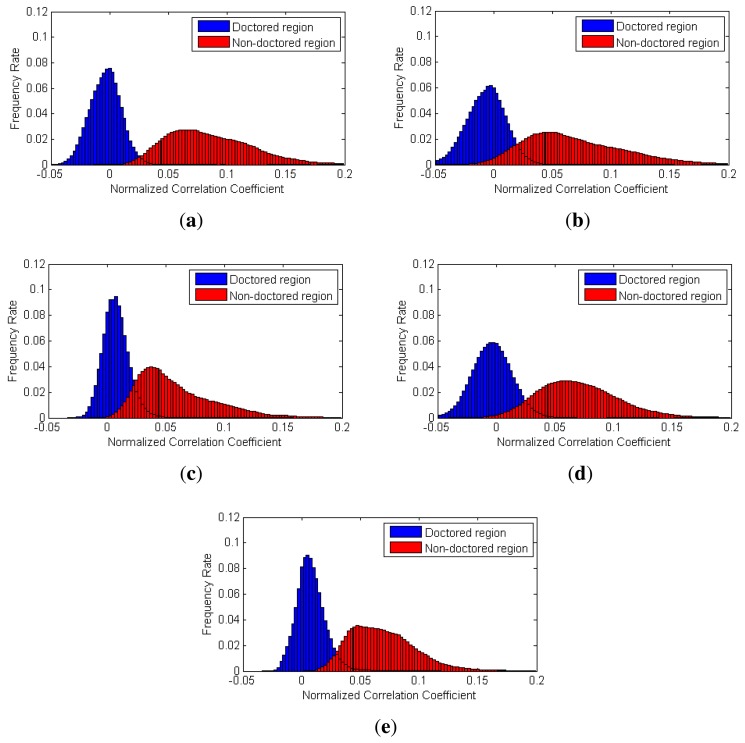
Histograms of the NCC values in the static-scene video: (**a**) the proposed method; (**b**) Chen's method with a 64 × 64 window; (**c**) Chen's method with a 128 × 128 window; (**d**) Li's method with a 64 × 64 window; (**e**) Li's method with a 128 × 128 window.

Figure 12 shows the histograms of the NCC values calculated by each method for the static-scene videos. We can observe that the NCC values of the doctored and non-doctored regions, which were extracted from the proposed method, are better separated than the NCC values of the other methods.

**Figure 13.**
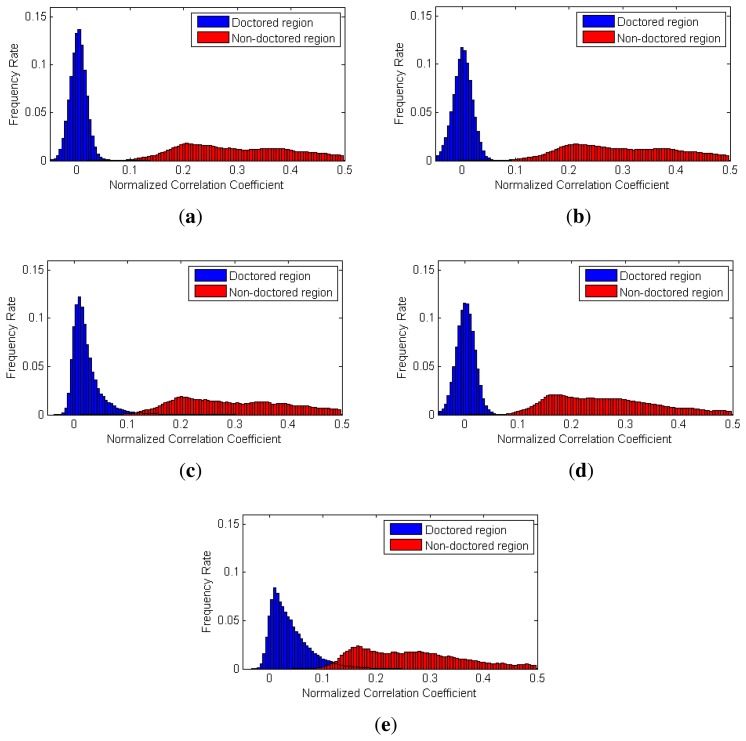
Histograms of the NCC values in the dynamic-scene video: (**a**) the proposed method; (**b**) Chen's method with a 64 × 64 window; (**c**) Chen's method with a 128 × 128 window; (**d**) Li's method with a 64 × 64 window; (**e**) Li's method with a 128 × 128 window.

Figure 13 shows the histograms of the NCC values calculated by each method for dynamic-scene videos. As contaminated SPN values rarely exist in the dynamic-scene video, correlation values from non-doctored regions are normally higher than those in the static-scene video. Thus, as shown in Figure 13, the NCC values of the doctored and non-doctored regions extracted by the proposed method, Chen's method and Li's method with a 64 × 64 window were successfully separated. However, Chen's method and Li's method with a 128 × 128 window sometimes had higher NCC values in the doctored region than those of the non-doctored regions, since the size of the local searching window was larger than the size of the forged region for some test videos.

**Figure 14.**
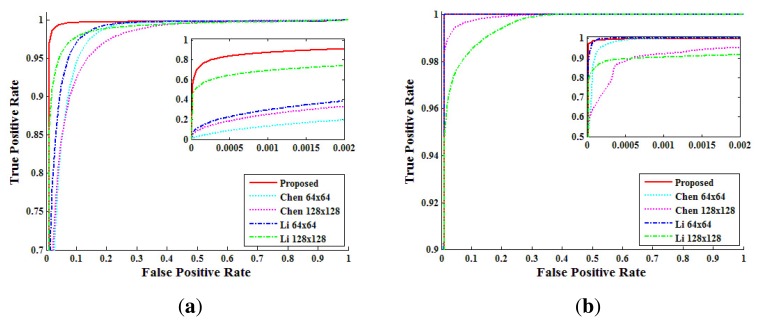
receiver-operating-characteristic (ROC) curves of each method for detecting forged pixels: (**a**) static-scene video; (**b**) dynamic-scene video.

Second, from the above analysis, we compared the performance of each method by receiver-operating-characteristic (ROC) curves. The pixel level true positive rate (TPR*_pixel_*) versus the false positive rate (FPR*_pixel_*) was calculated from the NCC values by changing the threshold. Figure 14 shows the pixel level ROC curves according to each method. In the static-scene video, the proposed method detected forgery with higher accuracy than both Chen's method and Li's method. In the dynamic-scene video, the proposed method, Chen's method and Li's method with a 64 × 64 window detected forgery in FPR*_pixel_* < 10^−2^ with TPR*_pixel_* higher than 99%. However, Chen's method and Li's method with a 128 × 128 window detected forgery with lower TPR*_pixel_* than the other methods, because the size of the detection block was larger than that of the forged region for some test videos. From the analysis of Figure 14, we set the thresholds, τ*_pixel_*, of the proposed method for static scene and dynamic scene videos as 0.0092 and 0.0815, respectively.

Finally, we analyze the computational complexity of each method. Three detectors determine forged pixels by computing NCC values between local blocks of test SPN and the corresponding reference SPN blocks as Equation (21). Computing an NCC value for a pixel takes *θ*(*log*(*BS* × *BS*)) by fast Fourier Transform. Therefore, the required time complexity is as below.

(24) θ(log(BS×BS)×M×N)

where *M, N* and *BS* are the height and width of an investigated video and the block size of the local searching window, respectively. Equation (24) reveals that the computational complexity is mostly determined by the block size of the local searching window. Therefore, the computational complexity of each method can be compared by the size of the local search window. We measured the average size of the local searching window of the proposed method, since our method adaptively determines the size of the window as shown in Equation (19). The average block size of the proposed method was 73.21 × 73.21, which was a little faster than Chen's and Li's method with 128 × 128 blocks and a little slower than those of 64 × 64 blocks.

### 6.4. Partial Manipulation Detection Experiments at the Video Level

After determining the forged region at a pixel level as depicted in Section 6.3, we finally determined whether or not the investigated videos were forged in this section. More specifically, we measured the performance of partial manipulation detection among three detectors (the proposed, Chen's and Li's approaches) at the video level. For that purpose, 240 unforged videos were created by compressing 120 sample videos with H.264 with quality factors of 80 and 100. Then, 1,440 forged videos were created as explained in Section 6.3. For all test videos, we calculated the NCC values of each method and generated forgery map *Z* in Equation (22) by thresholding the NCC values using τ*_pixel_*.

From the map, *Z*, we can determine whether the test video was forged or not by comparing the number of forged pixels with threshold τ_v_*_ideo_* in Equation (23). If the number of forged pixels in a test video is more than τ*_video_*, the investigated video is determined to be forged; otherwise, it is determined to not be forged. By choosing various τ*_video_*, we generated video-level ROC curves of a true positive rate at the video level (TPR*_video_*) versus a false positive rate at the video level (FPR*_video_*).

**Figure 15.**
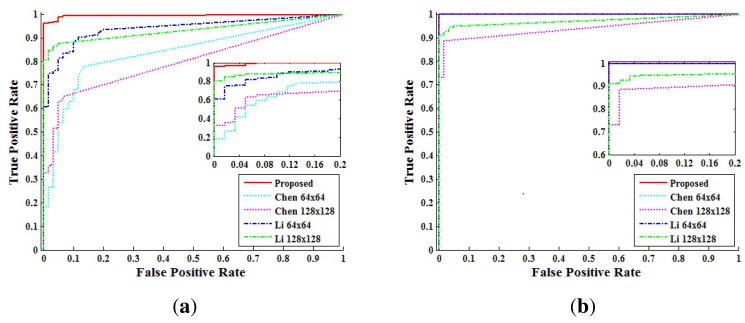
ROC curves of each method for detecting forged videos: (**a**) static-scene video; (**b**) dynamic-scene video.

Figure 15 shows the ROC curves at the video level for each method. The ROC curves confirm that the proposed method detects forgery with a low FPR*_video_* while achieving higher accuracy than Chen's method and Li's method, especially for the static-scene.

For a more detailed comparison with each method, we set the τ*_video_* as FPR*_video_* = 10^−2^ and calculated the forgery detection accuracy. Table 5 presents the detection accuracy of each method at FPR*_video_* = 10^−2^. For static-scene videos, Chen's method and Li's method with a 64 × 64 window had low accuracy. Li's method with a 128×128 window detected forgery with over 90% accuracy, only if the size of the doctored region was bigger than 140 × 140. On the other hand, the proposed method detected forgeries with over 90% accuracy regardless of the size of the doctored region. In the dynamic-scene videos, the proposed method and Li's method with a 64 × 64 window detected forgery with an accuracy of 100% in all doctored region sizes.

## 7. Conclusion

With the growth of the digital imaging industry, surveillance cameras are widely used throughout our society. As a result, in many court cases, surveillance videos are used as court evidence. At the same time, forgeries of surveillance videos are rapidly increasing. In this paper, we focused on a forensic technique to detect altered surveillance videos. Two types of forgeries, called upscale-crop forgery and partial manipulation, were identified by the proposed method based on sensor pattern noise (SPN). We exploited the scaling tolerance of a minimum average correlation energy Mellin radial harmonic (MACE-MRH) correlation filter to reliably unveil traces of upscale-crop forgery of videos. By excluding high-frequency components of the investigated video and adaptively selecting the size of the window block, the proposed method effectively detected partially manipulated regions in static-scene videos. Empirical evidence from a large database of test videos supported the superior performance of our techniques regardless of RGB/infrared video, dynamic-/static-scene video and compression.

## Figures and Tables

**Table 1.** Surveillance camera models used in the experiments.

**Brand**	**Model**	**Brand**	**Model**
IDIS	IDC-414BR	IDIS	407BR-B
CNB	B2760N	HUVIRON	NT8C
DALS	DS-P110/M430	CNB	XCL-20S KC3
DALS	C160(mini IR)	DALS	DSV100IR/M434
IDIS	407BR-A	HUVIRON	P461/M864N

**Table 2.** The normalized correlation coefficient (NCC) values between selected blocks of test SPN extracted from Figure 9a and the corresponding reference SPN; two blocks from the textured region (blue) and the other two blocks from the smooth region (red). Breakdown by method and block.

	**Chen**	**Li**	**Proposed**
64 × 64 Blue block	−0.003455	0.049101	0.089420
128 × 128 Blue block	0.046220	0.082426	0.094428
64 × 64 Red block	0.120120	0.122120	0.120120
128 × 128 Red block	0.129220	0.118833	0.129361

**Table 3.** Detection accuracy (%) of upscale-crop forgery for varying scaling and quality factors.

	**Quality Factor**	**Scaling Factor**								**Average**

		**1.05**	**1.1**	**1.15**	**1.2**	**1.25**	**1.3**	**1.35**	**1.4**	
Proposed	100	100	100	100	100	100	100	100	100	100
	80	100	98.3	100	100	99.2	100	100	99.2	99.6
Mahdian	100	60.0	86.7	97.5	100	98.3	100	100	96.7	92.4
	80	44.2	70.8	73.3	80.8	70.8	78.3	85.8	84.2	73.5
Kirchner	100	51.7	91.7	99.2	100	99.2	100	86.7	100	91.1
	80	20.0	46.7	50.8	68.3	87.5	79.2	20.8	62.5	54.5

**Table 4.** Detection accuracy (%) of upscale-crop forgery, even after the intended partial manipulation of upscale-cropped videos; the breakdown by the size of the doctored region.

**Scaling factor**	**Method**	**Quality factor**	**Doctored region size**			

			**50×50**	**100×100**	**150×150**	**200×200**
1.15	Proposed	100	100	100	100	99.2
		80	100	100	100	99.2
	Mahdian	100	81.7	60.8	33.3	21.7
		80	37.5	30.0	12.5	8.3
	Kirchner	100	99.2	99.2	96.7	92.5
		80	49.2	46.7	40.0	31.7

1.3	Proposed	100	100	100	100	100
		80	100	100	98.3	98.3
	Mahdian	100	99.2	80.8	60.8	45.0
		80	59.2	45.0	28.3	14.2
	Kirchner	100	100	100	99.2	98.3
		80	74.2	70.8	65.8	47.5

**Table 5.** Detection accuracy (%) of partial manipulation at FPR*_video_* = 10^−2^ according to the size of the doctored region.

**Video type**	**Method**	**Doctored region size**					

		**150×150**	**140×140**	**130×130**	**120×120**	**110×110**	**100×100**
Static-scene	Proposed	100	97.5	94.2	96.7	95.0	94.2
	Chen 64×64	25.8	20.8	20.8	20.0	11.7	9.2
	Chen 128×128	64.2	44.2	30.8	26.7	19.2	10.0
	Li 64×64	68.3	66.7	60.8	59.2	59.2	50.0
	Li 128×128	97.5	91.7	86.7	84.2	66.7	55.8

Dynamic-scene	Proposed	100	100	100	100	100	100
	Chen 64×64	100	100	100	100	100	99.2
	Chen 128×128	100	96.7	83.3	66.7	48.3	43.3
	Li 64×64	100	100	100	100	100	100
	Li 128×128	100	100	100	97.5	84.2	64.2
